# Climate Change and Phenology: *Empoasca fabae* (Hemiptera: Cicadellidae) Migration and Severity of Impact

**DOI:** 10.1371/journal.pone.0124915

**Published:** 2015-05-13

**Authors:** Mitchell B. Baker, P. Dilip Venugopal, William O. Lamp

**Affiliations:** 1 Biology Department, Queens College of The City University of New York, 65–30 Kissena Boulevard, Flushing, New York, 11367, United States of America; 2 Department of Entomology, University of Maryland, 4112 Plant Sciences Building, College Park, Maryland, 20742, United States of America; Scientific Research Centre, Slovenian Academy of Sciences and Arts, SLOVENIA

## Abstract

Climate change can benefit individual species, but when pest species are enhanced by warmer temperatures agricultural productivity may be placed at greater risk. We analyzed the effects of temperature anomaly on arrival date and infestation severity of potato leafhopper, *Empoasca fabae* Harris, a classic new world long distance migrant, and a significant pest in several agricultural crops. We compiled *E*. *fabae* arrival dates and infestation severity data at different states in USA from existing literature reviews and agricultural extension records from 1951–2012, and examined the influence of temperature anomalies at each target state or overwintering range on the date of arrival and severity of infestation. Average *E*. *fabae* arrival date at different states reveal a clear trend along the south-north axis, with earliest arrival closest to the overwintering range. *E*. *fabae* arrival has advanced by 10 days over the last 62 years. *E*. *fabae* arrived earlier in warmer years in relation to each target state level temperature anomaly (3.0 days / °C increase in temperature anomaly). Increased temperature had a significant and positive effect on the severity of infestation, and arrival date had a marginal negative effect on severity. These relationships suggest that continued warming could advance the time of *E*. *fabae* colonization and increase their impact on affected crops.

## Introduction

Global surface temperature has increased by about 0.74°C in the 100 year period ending in 2005 [1] and the decade 2000–2009 was the warmest on record. There is consensus that most of the observed warming is due to human release of CO_2_ into the atmosphere [2]. The most recent United States Dept. of Agriculture Plant Hardiness Zone (the standard by which growers determine which plants are most likely to thrive at a location) map, which averages winter minimum temperatures from 1976–2005, has a modal increase of one full zone relative to the 1990 map that averages temperatures from 1976–1988 [3]. However, climate change has diverse effects that vary geographically, and not every species studied has accelerated its phenology in response to recent warming [4]. Increase in temperatures due to climate change may increase the risk of invasion by migrant agricultural pests [5] and long distance migration of insect pests is likely affected by climate change. Here, we relate a 62-year record of the phenology of migration by *Empoasca fabae* Harris, Hemiptera: Cicadellidae, also known as potato leafhopper, to the temperature anomalies associated with climate change.

Climate change affects insect population dynamics such that some species gain in prominence while others become inconspicuous. Rising temperatures directly contribute to the acceleration of phenologies in insects [6], and phenologies of several species of Lepidoptera, Aphidoidea, and Odonata [7,8] have accelerated in recent decades, though behavioral temperature regulation could buffer climate effects [9]. Earlier colonization and more rapid insect development generally increase population growth. Temperature can also affect population dynamics and geographic range of any interacting species, including host-plants, natural enemies, and parasites [5,10,11], with potentially positive or negative effects on any given species. For long distance migratory insects whose host plants are available prior to arrival, the expectation is that earlier migration will increase the severity and economic impact of infestation. Because historical records are often kept for economically significant insect pest species, they can serve as models for understanding the response of migratory species to climate change. Moreover, climate change may exacerbate agricultural problems associated with migratory pests.

Climate change may affect migratory species differently than non-migrants. Coastal and subtropical areas are expected to experience less warming than temperate and polar areas[1], and migrants avoid winter temperatures, which have risen more than summer temperatures[12]. Migration complicates the already-challenging question of how climate change alters synchrony of herbivores, host plants and natural enemies [13–15]. While the phenology of many insects has advanced with warming [7,16], the response of long-distance migratory insects and vertebrates has been less well studied than that of non-migratory insects or vertebrates [17].

*E*. *fabae* is a classic example of insect pest migration in eastern North America [18,19]. It is multivoltine with populations continuously reproducing with overlapping generations [20]. It feeds and reproduces on over 200 species of plants, including at least 26 plant families [21]. It is a key pest on a number of its numerous host plants, ranging from potato (*Solanum tuberosum* L.) to alfalfa (*Medicago sativa* L.), and to red maple trees (*Acer rubrum* L.) [19, 22–24]. Especially, as a key pest of alfalfa in the northcentral and northeastern United States, *E*. *fabae* results in economic yield losses of up to $66 / ha [25]. Rather than primarily affecting hosts through vectoring plant disease, *E*. *fabae* feeding initiates a cascade of biochemical and physiological changes in its host [26] that impacts agricultural producers through reduced yield or quality of plants.

Evidence suggests that *E*. *fabae* is a circular migrant [27,28]. Adults are transported by prevailing winds to the south in the fall in reproductive diapause, and overwinter in southern pine forests [29,30]. In January-February, adults move from overwintering hosts to herbaceous legumes and deciduous trees to reproduce [29]. Adults of this generation, and of subsequent generations, move northward with synoptic weather patterns [31,32] and colonize north-central and north-eastern United States, and eastern Canada [33].

Climate change may alter the migration phenology of *E*. *fabae*. Since the timing of migration may impact population ecology of *E*. *fabae* and its severity on crops, we expect climate change will impact Integrated Pest Management (IPM) programs designed to manage the pest. In this study, using historical data on *E*. *fabae* arrival time and severity data compiled from several sources, we sought to: 1) examine the trend of delayed or earlier *E*. *fabae* arrival in continental United States over a 62-year time period, 2) investigate the effect of temperature anomaly on earliest reported *E*. *fabae* arrival times, and 3) investigate the effect of arrival day and temperature anomalies on severity of *E*. *fabae* on crops. To our knowledge this research represents the first such effort examining the influence of climate change on cicadellid leafhopper migration.

## Methods

### Ethics Statement

Data for this study were derived primarily from literature, and climatic data were obtained from freely available resources (National Oceanic and Atmospheric Administration (NOAA)—National Climatic Data Center). No endangered or protected species were involved in the study.

### First arrival dates of E. fabae and severity of impact

The first reported arrival dates of *E*. *fabae* in various states in the breeding range across the United States were compiled from existing literature and direct reports, and the infestation severity levels were obtained from an earlier published compilation. Arrival data prior to 1998 and severity data were from obtained from several sources gathered or compiled by Maredia et al. [34]. That study combined published studies of arrival date and severity [18,31,34] with reports from a network of several cooperators in central and northeastern US states. Severity was rated on a 1–5 scale (5 denoting most severe) based on percent of (primarily alfalfa) crop above economic impact threshold (details in [34]). Because of the reduced impact of *E*. *fabae* in alfalfa in this century, recent severity data were sparse and arrival dates have been recorded less frequently as well. Arrival dates for years since 1997 were obtained from published extension newsletters or cooperator records in seven states (see S1 Table). We note that there could be systematic differences between Maredia *et al*. [34] and our study in the network of monitoring and reporting methods for various sources. In particular, Illinois records since 1997 were taken from the phrase “are active” in a published pest bulletin, while the earlier records were collected by G. Decker, S. J. Robertson, and E. J. Armbrust [34]. Pennsylvania and Illinois arrival dates were notably different for years since 1997, and were divided into early (<1997) and late (>1997) periods in the compiled dataset. We obtained 342 records in 19 states for the first arrival of *E*. *fabae* from 1951–2012 (S1 Dataset). In addition, we obtained 196 records from 18 states for the severity of damage by *E*. *fabae* from 1951–1997. In many of the states with high number of data records, *E*. *fabae* is particularly recognized as an economically important crop pest.

For the study period, temperature anomaly (observed difference in temperature as compared to the average temperature for the reference period 1901–2000) data were obtained from NOAA—National Climatic Data Center (http://www.ncdc.noaa.gov/cag/time-series). For each study year, temperature anomaly values were calculated for each target *E*. *fabae* arrival state (19 states), and as the averages of *E*. *fabae* overwintering states (6 states with more than half their area in overwintering zones; see [29]), during winter (January through March), spring (April through June) and both seasons (January through June). Thus, six separate temperature anomaly variables were tabulated to use as explanatory variables for statistical analyses relating temperature anomalies to *E*. *fabae* arrival date. For severity of infestation, values were obtained by averaging temperature data during summer (May—July) for each target state, and then used as a predictor variable.

### Statistical Analyses

#### First arrival dates of *E*. *fabae*

Four data points (1.2% of total observations) on *E*. *fabae* arrival dates that were extreme outliers (Julian days 82, 86,105, 199) were removed from the data set. All statistical analyses were performed on the reduced dataset using linear mixed model (LMM) analysis with state as random factor to control for repeated measurement [35], as state was the unit of measurement for the arrival day data repeatedly collected over years. The trend of change in arrival dates over the 62-year time period was examined using LMMs based on restricted maximum likelihood (REML), with arrival date as the response variable, year as the fixed effect and state as the random effect.

#### Temperature influence on arrival day

The influence of temperature anomaly on arrival dates was analyzed through LMMs based on REML. For the LMM, arrival day was the response variable, temperature anomaly was a fixed effect, and states were used as a random effect to account for repeated measurement [35]. We computed multiple LMMs each with one of the six different temperature anomaly variables tabulated as the explanatory variable. We identified the best model based on both Akaike Information Criteria (AIC) and Bayesian Information Criteria (BIC) values.

The significance of fixed effects in the LMMs was determined through Wald t-test. Also, a likelihood-ratio based pseudo-R^2^ value [36] was calculated for each model as a measure of the proportion of total variance explained. Diagnostic plots of the models visualizing within-group residuals (standardized residuals Vs fitted values, normal Q-Q plots, histograms of residuals) and estimated random effects (normal Q-Q plots and pairs-scatter plot matrix) were used to assess model appropriateness (see [35]; pgs. 174–197).

#### Severity of impact

The effect of arrival date, as well as of temperature anomaly, on the severity of *E*. *fabae* infestation was analyzed using cumulative link mixed models (CLMM). CLMM was selected because it is appropriate for mixed effects models with ordinal data. CLMMs were performed on the overall data with severity of infestation as ordinal response factor, state as random effect accounting for repeated measurement, and interaction effect or individual effect of date of arrival and temperature anomaly as fixed effects. The interaction of date of arrival and temperature anomaly was tested prior to testing the individual influences of date of arrival and temperature anomaly. The significance of the fixed effects was determined through a likelihood ratio test. The proportional odds assumption for the CLMM models was verified to ensure model appropriateness (see [37] for more details).

LMMs were performed with package ‘nlme’ ([38]; v 3.1–102) and pseudo-R^2^ values were calculated using package ‘MuMIn’ ([39]; v 1.10.0). LMMs estimated coefficients were extracted and plotted using package ‘effects’ [40]; v 2.2–4) and CLMM were performed using package ‘ordinal’ ([41]; v 2014.12–23), all in R program [42].

## Results

### First arrival dates of E. fabae

**Fig 1:**
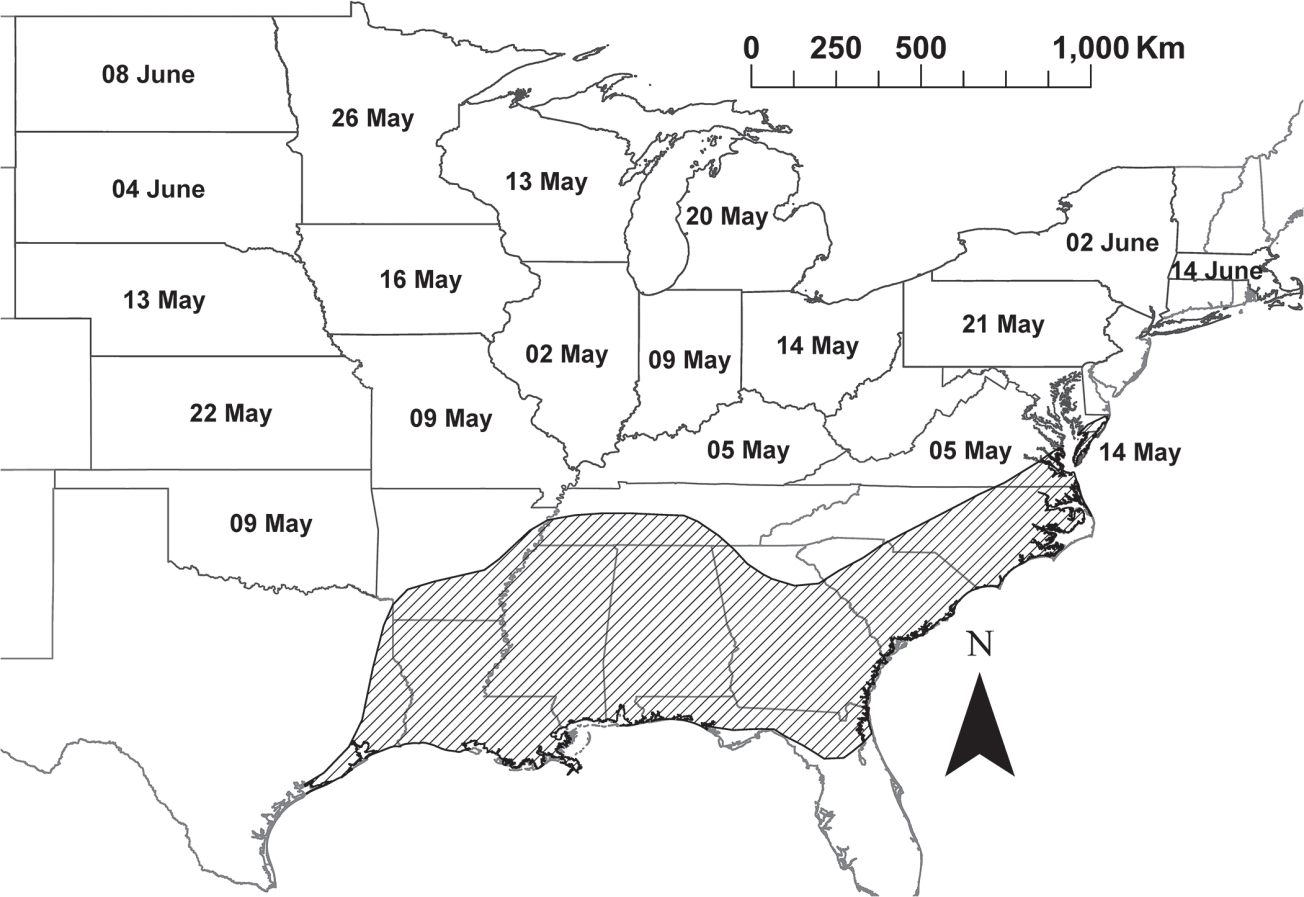
Average arrival date of *Empoasca fabae* at different states across continental United States during 1951–2012, and the overwintering range (stripe shaded region; adapted from Taylor and Shields [29]). Map was generated with spatial data on administrative state boundaries available freely through United States Census Bureau (https://www.census.gov/geo/maps-data/data/tiger-line.html).

**Fig 2:**
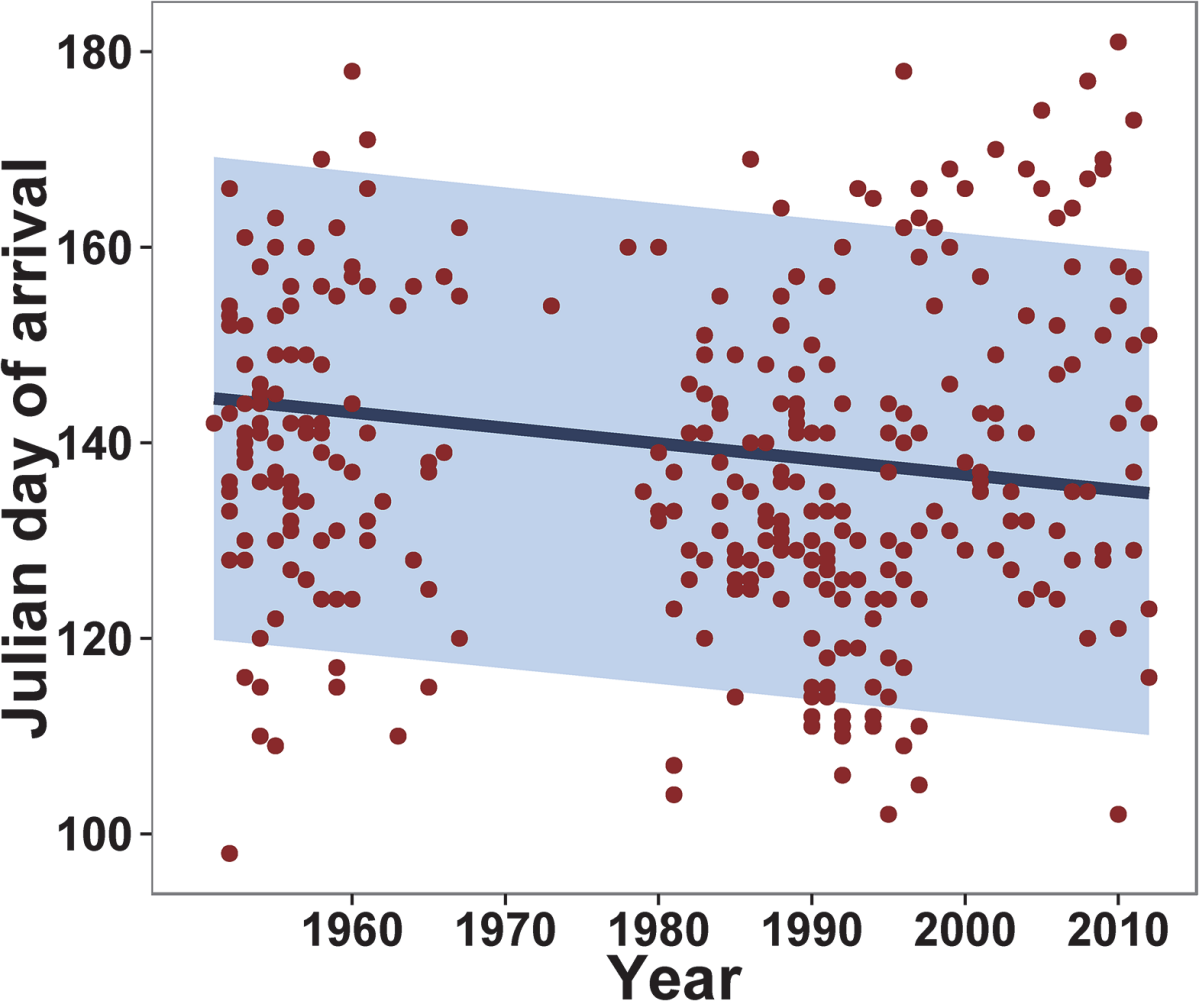
Trends in arrival date (Julian day) of *Empoasca fabae* in United States during 1951–2012 estimated through LMM. The straight line represents the slope and the extent of shaded region represents lower and upper 95% confidence intervals as estimated by the LMM, and the points represent raw data.

The average annual arrival date of *E*. *fabae* at different states in contiguous US, based on the compiled data, is provided in Table 1. First reported average arrival date increased along a south to north axis, corresponding to the distance from the overwintering sources in the southern part of USA (Fig 1). LMMs that examined trend of arrival demonstrated, after accounting for the state effect, a weak but significant negative association between year and arrival date (*b* = - 0.159, SE = 0.04, df = 316, Wald t = -3.86, *P* < 0.001). The model estimated that *E*. *fabae* adults arrived 0.16 days earlier with each yearly increase, or 9.7 days over 62 years (Fig 2).

**Table 1:** Summary of *Empoasca fabae* arrival and severity data across continental United States compiled for the study.

State	Arrival data sample size	Mean arrival day ± SD	Arrival data year range	Severity data sample size	Mean severity ± SD	Severity data year range
Illinois	24	122 ± 13.9	1952–1997	14	2.7 ± 1.1	1952–1992
Illinois (>1997)	7	150 ± 14.0	2004–2012	-	-	-
Indiana	9	129 ± 17.8	1952–1992	5	3.2 ± 1.1	1952–1992
Iowa	7	136 ± 12.6	1952–1992	3	4 ± 1.0	1953–1992
Kansas	4	142 ± 10.3	1952–1956	3	1.7 ± 0.6	1954–1956
Kentucky	17	125 ± 7.4	1980–1997	12	1.8 ± 1.1	1981–1992
Maryland	33	134 ± 13.3	1955–2012	18	3.3 ± 0.9	1955–1997
Massachusetts	16	165 ± 8.2	1991–2012	-	-	-
Michigan	29	140 ± 13.5	1953–2003	19	2.7 ± 1.1	1954–1997
Minnesota	31	146 ± 11.8	1952–2011	19	2.6 ± 1.2	1952–1997
Missouri	18	129 ± 13.0	1952–1995	14	2.3 ± 1.2	1953–1997
Nebraska	12	133 ± 121.3	1952–1989	9	2.8 ± 1.3	1952–1988
New York	11	153 ± 13.3	1953–1992	7	2.7 ± 1.7	1953–1992
North Dakota	9	159 ± 18.0	1953–1961	7	2.4 ± 1.1	1955–1961
Ohio	23	134 ± 11.4	1952–1997	14	2.6 ± 1.3	1952–1992
Oklahoma	23	129 ± 13.9	1952–1997	21	1.3 ± 0.5	1954–1997
Pennsylvania	6	141 ± 9.0	1955–1992	5	1.4 ± 0.5	1955–1992
Pennsylvania (>1997)	8	160 ± 10.1	1997–2012	-	-	-
South Dakota	7	155 ± 13.6	1952–1960	7	2.3 ± 1.0	1952–1960
Virginia	6	125 ± 14.0	1953–1992	5	3.4 ± 0.9	1953–1992
Wisconsin	42	133 ± 12.0	1951–2012	14	2.9 ± 0.9	1951–1992

The mean values provided are Julian day of arrival and severity of infestation on a scale of (1 = low to 5 = high) and States are arranged alphabetically.

### Temperature influence on arrival day

**Fig 3:**
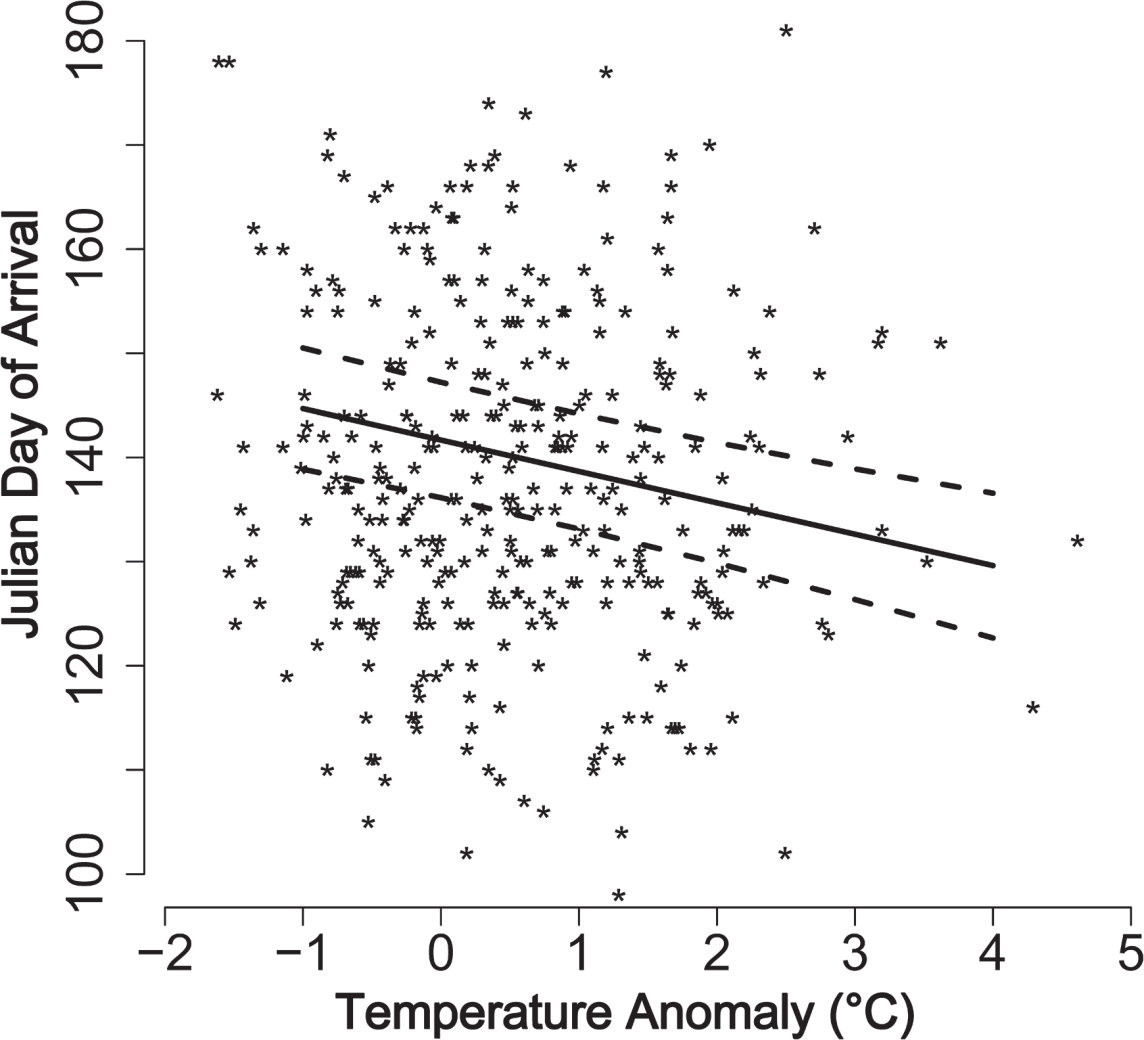
Arrival date (Julian day) of *Empoasca fabae* in relation to temperature anomaly in United States during 1951–2012, estimated through LMM. The straight lines represent the slope and dotted lines represent the lower and upper 95% confidence intervals as estimated by the LMM, and the points represents raw data.

LMM with temperature anomaly calculated for the target states across both winter and summer (January through June), was selected based on AIC and BIC values (see Table 2). Arrival date (in Julian days) of *E*. *fabae* was significantly influenced negatively by temperature anomaly at each individual target states during January—June (141.68 (±2.8)– 3.01 (±0.63) temperature anomaly, Wald-t = -4.80, d. f. = 316, *P* < 0.001). *E*. *fabae* arrived three days earlier in relation to 1°C increase in temperature anomaly. The anomaly accounted for a third of the variation in arrival day (pseudo-R^2^ = 0.37; Fig 3).

**Table 2:** Summary of LMM results analyzing the influence of temperature anomaly on the overall earliest arrival day of *Empoasca fabae* in the United States during 1951–2012.

Temperature anomaly data range	Data months	Estimate	SE	pval	Pseudo—R^2^	AIC	BIC	logLik
Individual target states	January—June	-3.01	0.63	<0.001	0.37	2687.3	2702.6	-1339.6
Individual target states	January—April	-1.68	0.39	<0.001	0.37	2692.3	2707.6	-1342.2
Overwintering states	January—June	-2.82	0.87	<0.001	0.35	2698.3	2713.6	-1345.2
Overwintering states	January—April	-1.38	0.51	<0.001	0.34	2702.4	2717.7	-1347.2
Individual target states	April—June	-1.61	0.65	0.0136	0.34	2703.2	2718.5	-1347.6
Overwintering states	April—June	-1.63	0.90	0.07	0.33	2705.4	2720.7	-1348.7

For each LMM, temperature anomaly was calculated differently as averages based on distributional range and seasons (see methods for details on overwintering and target states). Models are arranged in increasing values of AIC and BIC (Akaike information and Bayesian information criteria respectively).

### Severity of impact

CLMM revealed a non-significant interaction term (Z value 1.70, *P* = 0.090) of the interaction of arrival day and temperature anomaly on infestation severity of *E*. *fabae*. CLMM with only arrival day as predictor variable was marginally significant based on a likelihood ratio test (χ^2^ = - 3.73, d.f. = 1, *P* = 0.053). Also, results showed a mild yet positive association between temperature anomaly and severity levels with 0.54 (±0.17) times greater overall odds for higher infestation with increasing state temperature anomaly which was significant in a likelihood ratio test (χ^2^ = 11.14, d.f. = 1, *P* < 0.001). In particular, the odds ratio for higher severity levels increased with temperature anomaly with 1.5 (± 0.30) times greater odds between severity scales 3 and 4, and 3.7 (±0.45) times greater odds between severity levels 4 and 5.

## Discussion

Climate change may aggravate migratory insect pest impacts in agriculture. We found a trend of earlier arrival over a 62-year time period ending in 2012 (9.7 days), with a significant negative relationship between mean annual temperature anomaly and arrival of *E*. *fabae* in its summer range. Results also revealed a significant positive effect of temperature on *E*. *fabae* severity of infestation, and a marginally significant negative effect of arrival date on potato leafhopper severity of infestation. While future impacts are very challenging to anticipate due to interactions among many factors influencing insect population dynamics, these results suggest an increasingly earlier time of colonization by *E*. *fabae* with continued warming, and that severity of infestation may also increase with warming.

The relationship between temperature and arrival date, -3.0 days °C^-1^ over all states, is within the range observed in other insects studied. The best studied systems to date are the Aphidoidea (-10 to -14 days °C^-1^ [43]; 0.611±0.11 days year^-1^ [44]) and Lepidoptera (~7 days °C^-1^ [45]; -2 to -10 days °C^-1^ [46]) with a few other cases including *Apis mellifera* L. (-5.5 days °C^-1^), *Leptinotarsa decemlineata* (Say) (-5.8 days °C^-1^), *Bactrocera oleae* (Rossi) (-5.2 days °C^-1^ [47]), Odonata (-3.4 days °C^-1^ [48]), and several species including Cicadidae and Orthoptera (-2.7 days °C^-1^ [16]). Predicting future arrival dates in *E*. *fabae* is challenging because of imprecise knowledge of the location of source populations and proximate causes of movement and settling.

The overwintering range of *E*. *fabae* extends north of the Gulf States [29,49], but it is not known which overwintering populations contribute most to the migration into northern states. Temperature effects on migration are diverse along the overwintering range, based on the best current understanding of *E*. *fabae* overwintering phenology. After overwintering on evergreens in a non-reproductive state, a largely female population shifts to leguminous hosts and deciduous trees in early spring [30]. It is unknown the degree to which indirect cues, such as photoperiod, vs. direct cues, such as population density or availability or quality of hosts, stimulate migration [50]. Increased temperature probably allows earlier migration through accelerated development of legumes and spring leafhopper generations. In addition, earlier stress or senescence and harvest of spring host plants may lead to migration. Thus, *E*. *fabae* migration may not be limited solely by readiness of spring migrants.

Temperature can operate on several processes necessary for *E*. *fabae* colonization of spring and summer ranges. It could advance the timing of favorable synoptic weather systems to carry migrants north. Literature on changes in the timing of synoptic weather systems and climate change is sparse. Kossin [51] found that the start of the north Atlantic hurricane season has been advancing, but these synoptic storm systems form later in the summer. Hondula and Davis [52] observed a decrease in the frequency of low-pressure transition days with increasing temperature in the Midwest, suggesting fewer southerly winds available for migration. Higher temperatures could shift the northern limit of the overwintering range and shorten migratory distances, allowing smaller scale and shorter weather patterns to suffice for migration. Finally, increased temperatures could advance the timing of favorable conditions (including host plant development) for colonists of northern states. *E*. *fabae* was detected on yellow sticky cards following two favorable weather systems for migration before they were widely detected using sweep nets later in the spring [53], suggesting migratory events sometimes precede host colonization.

Annual mean global and national temperatures have increased most consistently and clearly since 1980. Yet, there does not appear to be an increase in the advance of reported occurrence since 1980 (see Fig 2). In our study, the advancement of arrival time over the 62-year time period ending 2012 (~10 days) was greater than that predicted by temperature (3 days earlier arrival / °C increase in anomaly), but visually much of the advancement in arrival (as seen in Fig 2) precedes the greatest warming. The inconsistency between the response of *E*. *fabae* phenology to temperature and the observed trends over time is similar to an existing report for other insect species [16]. In that study the pattern was attributed to confounding factors from declining populations, or development and urbanization of habitats over the same time period. In our example a decline of *E*. *fabae* pest status, due to resistant alfalfa varieties [54] and incidental suppression in potato by systemic neonicotinoid insecticides targeted at Colorado potato beetle [55], has reduced search effort and the ability to detect early spring colonizers. It is also possible that factors other than temperature limit migration or colonization and have opposed or limited any effect during the last decade.

Ultimately changes in phenology due to warming are ecologically significant if they affect population size and interactions with other species. Historical records of infestation severity offer an opportunity to test both the ecological impact of climate effects on phenology, as well as phenological effects on abundance. For example, Cocu *et al*. [43] found a strong effect of mild winters on early migration in the aphid, *Myzus persicae* (Sulz.), which was associated with highly damaging outbreaks. We found a direct influence of temperature anomaly on infestation severity, and a marginally significant negative relationship between arrival date and severity. An earlier analysis using subsets (three states and two regions) of the same severity data did not find the predicted correlation between first occurrence date and infestation severity [34]. However, we agree with Maredia *et al*. [34] that many factors besides arrival date contribute to severity. Our results indicate that temperature during the growing season has a greater impact on severity than arrival time. Precipitation [56] and agricultural practices [57,58] also likely have large effects.

The degree to which preparedness to migrate, suitable weather systems, or readiness to support migratory populations after colonization pose the greatest limits to migratory species [59,60] is a continuing debate to which climate-induced population changes should contribute greatly. In turn, knowledge of the limiting factors on migratory species is needed to make any predictions about the effects of changing climate and how to manage economically important migrants. The historical records often available for agricultural pests are a valuable resource for predicting and testing climate effects. Continued and focused sampling effort is needed to build on and take advantage of those records.

## Supporting Information

S1 Table Sources for data on potato leafhopper arrival date since 1997. (DOCX)

S1 Dataset Data on yearly arrival date (Julian days) and severity of impact (1–5; 5 most severe) of *Empoasca fabae* in the United States. Temperature anomaly (°C) data for overwintering range and target states for winter, spring, and both winter and spring are also provided.
(XLSX)
