# Supplementary material for: Climate Change and Phenology: Empoasca fabae (Hemiptera: Cicadellidae) Migration and Severity of Impact
Source: PLoS One. 2015 May 13;10(5):e0124915. doi: 10.1371/journal.pone.0124915 (PMC4430490; doi:10.1371/journal.pone.0124915)
Supplement: S1 Table — (DOCX) [file pone.0124915.s001.docx]

**Table S1:** Sources for data on potato leafhopper arrival date since 1997.

| **State** | **Source** | **Institution** | **Years** |
| --- | --- | --- | --- |
| Illinois | The Bulletin | University of Illinois | 2004-2012 |
| Maryland | Lamp (unpub. data) | University of MD | 1998-2012 |
| Massachusetts | Vegetable Notes Newsletter | University of Massachusetts | 1991-2012 |
| Michigan | DiFonzo 2005 | Michigan State University | 1998-2003 |
| Minnesota | IPM Stuff | University of Minnesota | 2001-2012 |
| Pennsylvania | Field Crop News | Penn State University | 1997-2012 |
| Wisconsin | Wisconsin Pest Bulletin | University of Wisconsin | 2001-2012 |

The Bulletin - <http://bulletin.ipm.illinois.edu/>

Vegetable Notes Newsletter - <http://extension.umass.edu/vegetable/publications/vegetable-notes-newsletter>

Michigan – Potato leafhoppers in Michigan. Chris DiFonzo 2005

University of Minnesota Southwest Research and Outreach Center, Lamberton MN - <http://swroc.cfans.umn.edu/ResearchandOutreach/PestManagement/index.htm>

Penn State Extension - <http://extension.psu.edu/plants/crops/news>

Wisconsin Pest Bulletin - <http://datcpservices.wisconsin.gov/pb/index.jsp>
